# Coordination of Polyploid Chromosome Replication with Cell Size and Growth in a Cyanobacterium

**DOI:** 10.1128/mBio.00510-19

**Published:** 2019-04-23

**Authors:** Ryudo Ohbayashi, Ai Nakamachi, Tetsuhiro S. Hatakeyama, Satoru Watanabe, Yu Kanesaki, Taku Chibazakura, Hirofumi Yoshikawa, Shin-ya Miyagishima

**Affiliations:** a Department of Cell Genetics, National Institute of Genetics, Mishima, Shizuoka, Japan; b Department of Bioscience, Tokyo University of Agriculture, Tokyo, Japan; c Department of Basic Science, University of Tokyo, Tokyo, Japan; d Genome Research Center, Tokyo University of Agriculture, Tokyo, Japan; e Department of Genetics, Graduate University for Advanced Studies (SOKENDAI), Mishima, Shizuoka, Japan; Goethe University; Harvard University

**Keywords:** DNA replication, DnaA, polyploidy, RpoC, cyanobacteria

## Abstract

Homologous chromosome number (ploidy) has diversified among bacteria, archaea, and eukaryotes over evolution. In bacteria, model organisms such as Escherichia coli possess a single chromosome encoding the entire genome during slow growth. In contrast, other bacteria, including cyanobacteria, maintain multiple copies of individual chromosomes (polyploid). Although a correlation between ploidy level and cell size has been observed in bacteria and eukaryotes, it is poorly understood how replication of multicopy chromosomes is regulated and how ploidy level is adjusted to cell size. In addition, the advantages conferred by polyploidy are largely unknown. Here we show that only one or a few multicopy chromosomes are replicated at once in the cyanobacterium Synechococcus elongatus and that this restriction depends on regulation of DnaA activity. Inhibiting the DnaA intrinsic ATPase activity in *S. elongatus* increased the number of replicating chromosomes and chromosome number per cell but did not affect cell growth. In contrast, when cell growth rate was increased or decreased, DnaA level, DnaA activity, and the number of replicating chromosomes also increased or decreased in parallel, resulting in nearly constant chromosome copy number per unit of cell volume at constant temperature. When chromosome copy number was increased by inhibition of DnaA ATPase activity or reduced culture temperature, cells exhibited greater resistance to UV light. Thus, it is suggested that the stepwise replication of the genome enables cyanobacteria to maintain nearly constant gene copy number per unit of cell volume and that multicopy chromosomes function as backup genetic information to compensate for genomic damage.

## Abstract (precis)

Polyploidy has evolved many times across the kingdom of life. The relationship between cell growth and chromosome replication in bacteria has been studied extensively in monoploid model organisms such as Escherichia coli but not in polyploid organisms. Our study of the polyploid cyanobacterium Synechococcus elongatus demonstrates that replicating chromosome number is restricted and regulated by DnaA to maintain a relatively stable gene copy number/cell volume ratio during cell growth. In addition, our results suggest that polyploidy confers resistance to UV, which damages DNA. This compensatory polyploidy is likely necessitated by photosynthesis, which requires sunlight and generates damaging reactive oxygen species, and may also explain how polyploid bacteria can adapt to extreme environments with high risk of DNA damage.

## INTRODUCTION

Chromosomal ploidy has diversified during evolution in all three domains of life, bacteria, archaea, and eukaryotes. Most eukaryotic organisms are diploid, but some plants are triploid or polyploid. Likewise, ploidy level varies in bacteria. Many bacteria adopted as models for cell biological studies, such as Escherichia coli and Bacillus subtilis, possess a single circular chromosome (genome) per cell (monoploid) during slow growth and become mero-origoploid during fast growth. In the case of Caulobacter crescentus, cells are monoploid irrespective of growth rate (1, 2). Alternatively, some bacteria maintain multiple chromosome copies per cell (polyploid) irrespective of growth rate as observed in cyanobacteria (3, 4), *Deinococcus* (5, 6), Thermus thermophilus (7), and symbiotic or parasitic bacteria such as *Buchnera* (8), *Neisseria* (9), *Borrelia* (10), and *Epulopiscium* (11). Chloroplasts and mitochondria, which evolved in eukaryotic cells from bacterial endosymbionts, are also polyploid. In addition, polyploidy has been reported in several lineages of archaea (12–16).

Although mechanisms that regulate polyploidy are not known, a positive association between ploidy level and cell size has been observed in a certain species of polyploid prokaryotes and eukaryotes. In polyploid prokaryotes such as the Gram-positive bacteria *Epulopiscium* spp. (17), the cyanobacterium Synechococcus elongatus (18), and the halophilic archaeon Halobacterium salinarum (13), larger cells possess more copies of individual chromosomes than smaller cells. Similarly, artificial polyploidy in the yeast Saccharomyces cerevisiae was associated with greater cell size (19). In plant epidermal cells as well, a positive association of ploidy with cell size has been observed (20). It has been assumed that cytoplasmic cell volume changes in a manner depending on ploidy level which regulates rate of ribosome biogenesis, gene expression level, or other factors in yeasts and plants (21, 22). However, the studies did not rule out the opposite possibility that cell volume regulates the ploidy level. Thus, it is unclear whether there is any causal relationship between ploidy level and cell size in polyploid organisms and, if so, whether cell growth increases ploidy level or increased ploidy level leads to increased cell size (21, 22). Further, the biological significance of this relationship remains obscure. Monoploidy is apparently advantageous for proliferation compared to polyploidy due to the lower energetic costs and reduced risks associated with DNA replication during cellular proliferation. Nevertheless, polyploidy has evolved many times in both prokaryotes and eukaryotes, implying that polyploidy confers certain survival advantages in specific environments.

Precise chromosomal DNA replication is essential for inheritance of advantageous genetic traits during proliferation. Molecular studies in model bacteria such as E. coli have shown that chromosome replication is tightly controlled mainly at the initiation stage of DNA replication. The initiator protein DnaA first binds to the replication origin (*oriC*) and then recruits components of the replisome (23, 24). DnaA AAA+ ATPase is highly conserved among bacteria. It binds both ATP and ADP, but only ATP-bound DnaA is capable of forming an oligomeric structure at the *oriC* region and initiating DNA replication. In E. coli, DnaA level is constant throughout cell cycle progression, whereas the ratio of ATP-DnaA to the total DnaA pool peaks just before chromosome replication in the cell cycle (25, 26). A similar regulatory mechanism for DnaA activity during the cell cycle has been observed in other bacteria (23, 27, 28). In contrast, DnaA activity in C. crescentus is regulated by a change in the total DnaA level (29, 30). It is believed that regulation of DnaA activity ensures chromosome replication once per cell cycle in monoploid bacteria, although the specific mode of regulation (i.e., DnaA level or ratio of ATP-DnaA to total DnaA) has diverged among species (31). In contrast to these monoploid bacteria, regulatory mechanisms for DNA replication are still poorly understood in polyploid bacteria.

Cyanobacteria are photosynthetic, and it has been reported that many species are polyploid (32–36). Recent studies in Synechococcus elongatus PCC 7942 have begun to reveal the regulatory mechanism of polyploid DNA replication in cyanobacteria. In *S*. *elongatus*, DNA replication is initiated at the *oriC* region and replication requires DnaA as in monoploid bacteria (37, 38). However, all multicopy chromosomes (four to six) are not replicated simultaneously; rather, only one or two chromosomes are replicated at any stage of the cell cycle (37–40). In addition, chromosomal copy number per cell changes and exhibits a positive and linear relation with cell size (18, 39, 40). Furthermore, despite the increase in cell volume, protein concentration remains constant (18); thus, it has been suggested that the increase in chromosomal copy number with growth allows the cell to maintain individual mRNA and protein concentrations (18). However, it is still unclear how replication of multiple chromosome copies is regulated so that only a few are replicated at once and how cell size and copy number are coordinated. In addition, it is not known whether all chromosomal copies contribute mRNA and protein production to maintain a constant protein concentration per cell.

Here we show that initiation of DNA replication and the number of replicating chromosomes are regulated by DnaA and that the rate of cell growth determines the number of replicating chromosomes by modulating DnaA level and activity in *S. elongatus*. In addition, it is shown that increasing the ploidy level also increases cellular resistance to UV irradiation, suggesting that possessing multicopy chromosomes allows the organism to cope with DNA damage.

## RESULTS

### Only a few chromosomal copies are replicated at once while genes are transcribed from all chromosomal copies in cyanobacteria.

Previous reports suggested that the number of replicating chromosomes is restricted to one (or in some case two) in *S*. *elongatus* (37–40). Zheng and O’Shea recently reported that chromosomal copy number and protein concentration per cell volume are maintained constant independently of cell size and growth rate (18) in *S*. *elongatus*. These observations imply that genes are transcribed from all chromosome copies to maintain a constant concentration of transcripts and proteins; however, the transcriptional activity at chromosomes was not examined.

**FIG 1:**
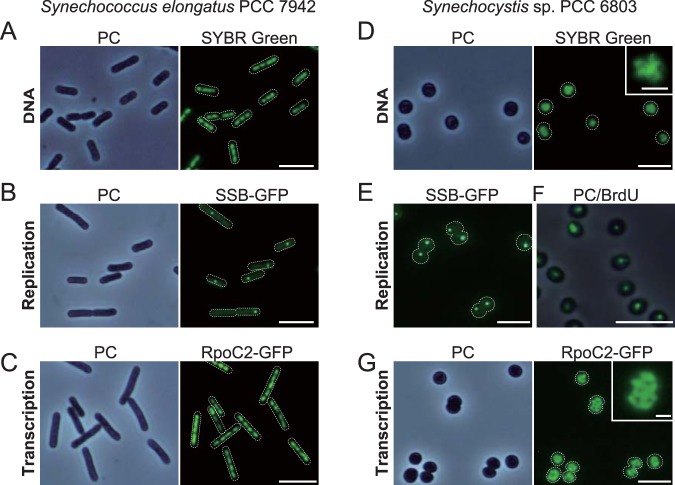
Replication and transcription of multicopy chromosomes in *S. elongatus* and *Synechocystis*. Mid-log-phase cultures were inoculated into fresh inorganic medium and cultured for 6 h under illumination at 70 µE m^−2^ s^−1^. (A and D) Microscopic images of SYBR Green-stained nucleoids in *S. elongatus* (A) and *Synechocystis* (D) cells. A magnified and high-contrast view of SYBR Green-stained *Synechocystis* cells is shown in the inset (D). The phase-contrast (PC) and SYBR Green-stained images of the same fields are also shown. (B and E) Fluorescence microscopic images of SSB-GFP expressers showing localization of SSB in *S. elongatus* (B) and *Synechocystis* (E) cells. PC and SSB-GFP images of the same field are shown for *S. elongatus*. (C and G) Fluorescence microscopic images of RpoC2-GFP expressers showing localization of RNA polymerase in *S. elongatus* (C) and *Synechocystis* (G) cells. A magnified view of RpoC2-GFP *Synechocystis* cells is shown in the inset (G). PC and RpoC2-GFP images of the same fields are also shown. (F) *Synechocystis* cells were labeled with BrdU for 1 h (from hour 5 to 6) and then fixed for immunofluorescence staining with the anti-BrdU antibody. The PC and immunofluorescence images are merged. Bars, 5 µm (1 µm for insets of panels D and G).

When *S. elongatus* cells were fixed and stained with a DNA-specific fluorescent dye, SYBR Green I, multiple (3 to 6) copies of chromosomes were observed aligned along the long axis of the cell (Fig. 1A). To examine transcriptional activities of multicopy chromosomes, we visualized replicating chromosomes and transcribing chromosomes in live *S. elongatus* cells. In addition, to assess the generality of the results, the same assays were applied to *Synechocystis* sp. strain PCC 6803 (here called *Synechocystis*), a model cyanobacterium phylogenetically distant from *S*. *elongatus* (41, 42). To this end, we expressed GFP-tagged single-strand binding protein (SSB; see Fig. S1 and S2 in the supplemental material), which is known to localize at replication forks (40, 43), and RNA polymerase beta subunit (RpoC2; Fig. S3 and S4). Consistent with previous reports (37, 39, 40), the majority of *S. elongatus* cells exhibited only one SSB focus (replication fork) per cell during growth under illumination (Fig. 1B). In contrast, RNA polymerase was observed on all chromosome copies (Fig. 1C).

10.1128/mBio.00510-19.2 FIG S1 Preparation of an *S*. *elongatus* SSB-GFP expresser. (A) The *gfp* ORF was fused with the *ssb* gene just before the stop codon. The construct was integrated into the chromosomal *ssb* locus by homologous recombination, and the gentamicin resistance gene (Gm^r^) was used as the selection marker for the transformant. (B) Insertion of the *gfp* and Gm^r^ genes into the chromosomal *ssb* locus of DnaA^WT^ and DnaA^R328H^ cells was confirmed by PCR using the primers indicated in panel A by arrows. The wild-type cell was used as a negative control. (C) Immunoblot analyses showing the expression of GFP-SSB in HA-DnaA^WT^ and HA-DnaA^R328H^ cells. HA-DnaA^WT^ and HA-DnaA^R328H^ cells without *GFP-SSB* were used as negative controls. Total proteins extracted from respective strains were subjected to analyses. GFP-SSB was detected with the anti-GFP antibody, and HA-DnaA was detected with the anti-HA antibody as a loading control. Download FIG S1, EPS file, 0.6 MB. Copyright © 2019 Ohbayashi et al. 2019 Ohbayashi et al. This content is distributed under the terms of the Creative Commons Attribution 4.0 International license.

10.1128/mBio.00510-19.3 FIG S2 Preparation of a *Synechocystis* SSB-GFP expresser. (A) The *gfp* ORF was fused with the *ssb* gene just before the stop codon. The construct was integrated into the *Synechocystis* chromosomal *ssb* locus by homologous recombination. The gentamicin resistance gene (Gm^r^) was used as the selection marker for the transformant. (B) Insertion of the *gfp* and Gm^r^ genes into the chromosomal *ssb* locus was confirmed by PCR using the primers indicated in panel A by arrows. The wild-type cell was used as a negative control. (C) Immunoblot analyses showing the expression of GFP-SSB in the transformant. The wild-type cell was used as a negative control. Total proteins extracted from the wild type and the transformant were subjected to analysis. GFP-SSB was detected with the anti-GFP antibody. As a loading control, Coomassie brilliant blue (CBB) staining of the protein samples resolved by SDS-PAGE is also shown. Download FIG S2, EPS file, 1.3 MB. Copyright © 2019 Ohbayashi et al. 2019 Ohbayashi et al. This content is distributed under the terms of the Creative Commons Attribution 4.0 International license.

10.1128/mBio.00510-19.4 FIG S3 Preparation of an *S. elongatus* RNA polymerase-GFP expresser. (A) The *gfp* ORF was fused with the *rpoC2* gene just before the stop codon. The construct was integrated into the chromosomal *rpoC2* locus by homologous recombination. The gentamicin resistance gene (Gm^r^) was used as a selection marker for the transformant. (B) Insertion of the *gfp* and Gm^r^ genes into the chromosomal *rpoC2* locus was confirmed by PCR using the primers indicated in panel A by arrows. The wild-type cell was used as a negative control. (C) Immunoblot analyses showing the expression of RpoC2-GFP in the transformant. The wild-type cell was used as a negative control. Total proteins extracted from the wild-type and the RpoC2-GFP cells were subjected to analysis. RpoC2-GFP was detected with the anti-GFP antibody. As a loading control, Coomassie brilliant blue (CBB) staining of the protein samples resolved by SDS-PAGE is also shown. Download FIG S3, EPS file, 2.3 MB. Copyright © 2019 Ohbayashi et al. 2019 Ohbayashi et al. This content is distributed under the terms of the Creative Commons Attribution 4.0 International license.

10.1128/mBio.00510-19.5 FIG S4 Preparation of a *Synechocystis* RNA polymerase-GFP expresser. (A) The *gfp* ORF was fused with the *rpoC2* gene just before the stop codon. The construct was integrated into the chromosomal *rpoC2* locus by homologous recombination. The spectinomycin resistance gene (Spec^r^) was used as the selection marker for the transformant. (B) Insertion of the *gfp* and Spec^r^ genes into the chromosomal *rpoC2* locus was confirmed by PCR using the primers indicated in panel A by arrows. The wild-type cell was used as a negative control. (C) Immunoblot analyses showing the expression of RpoC2-GFP in the transformant. The wild-type cell was used as a negative control. Total proteins extracted from the wild-type and RpoC2-GFP cells were subjected to analysis. RpoC2-GFP was detected with the anti-GFP antibody. As a loading control, Coomassie brilliant blue (CBB) staining of the protein samples resolved by SDS-PAGE is also shown. Download FIG S4, EPS file, 0.5 MB. Copyright © 2019 Ohbayashi et al. 2019 Ohbayashi et al. This content is distributed under the terms of the Creative Commons Attribution 4.0 International license.

*Synechocystis* possesses more chromosome copies (10 to 20) than *S*. *elongatus* (3 to 6) (3, 36). When genomic DNA of *Synechocystis* was stained with SYBR Green I, many small foci were observed in each cell (Fig. 1D). In contrast, SSB-GFP was detected at only one focus per cell (Fig. 1E). In a similar manner, only one chromosome per cell (or two in the case of dividing cells) incorporated 5-bromo-2′-deoxyuridine (BrdU), an analog of thymidine incorporated into newly synthesized DNA, during a 1-h observation period (Fig. 1F). In contrast, RNA polymerase localized on all chromosome copies (Fig. 1G). These results indicate that the number of replicating chromosomes is restricted to one or two while all chromosome copies are used as the templates for transcription both in *S. elongatus* and in *Synechocystis*.

### DnaA binding to the *oriC* region depends on photosynthesis.

**FIG 2:**
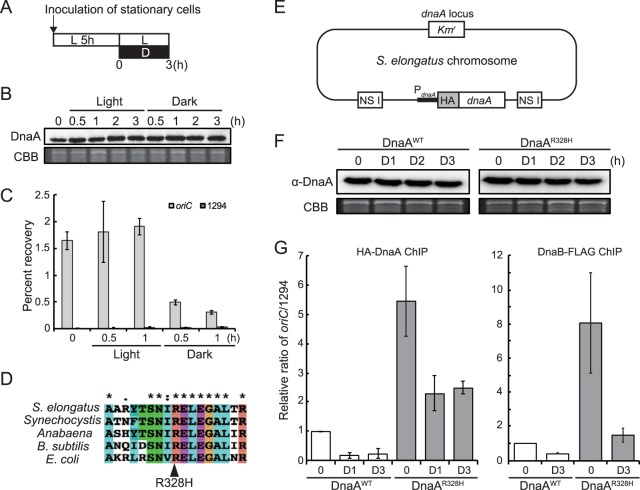
Effects of light and inhibition of DnaA intrinsic ATPase activity on affinity for the genomic *oriC* region. (A) Schematic diagram of the culture conditions for panels B, C, F, and G. Mid-log-phase cultures of the *S. elongatus* wild-type, DnaA^WT^, or DnaA^R328H^ strains were inoculated into fresh inorganic medium and cultured for 5 h under 70-µE m^−2^ s^−1^ illumination. At hour 0, one culture was kept under illumination and another culture was transferred to darkness. Both were cultivated for a further 3 h. (B) Immunoblot analysis showing the DnaA protein level under the light or dark condition. Proteins extracted from wild-type cells at the indicated time points were reacted with the anti-DnaA antibody (upper panel). Coomassie brilliant blue (CBB) staining of protein samples resolved by SDS-PAGE is shown in the lower panel as a gel loading control. (C) ChIP-qPCR analysis showing the affinity of DnaA for the *oriC* region under light and dark conditions. The DnaA-chromatin complex was immunoprecipitated with the anti-DnaA antibody. The samples were quantified by qPCR using primers specific to the *oriC* region (*oriC*, gray bar) and *Syf1294* gene (1294, dark gray bar). The percent recovery against the total amount of input DNA is indicated. (D) A partial amino acid sequence alignment of the DnaA proteins from *S*. *elongatus, Synechocystis, Anabaena* sp. strain PCC 7120 (*Anabaena*), Bacillus subtilis 168, and Escherichia col. The conserved arginine residue is indicated by the arrowhead. (E) Schematic diagram of the *S. elongatus* chromosome expressing HA-DnaA^WT^ (DnaA^WT^) or HA-DnaA^R328H^ (DnaA^R328H^). DNA encoding HA-DnaA^WT^ or HA-DnaA^R328H^ under the control of the *dnaA* promoter was introduced into the chromosomal neutral site I (NS I). The native *dnaA* gene was then deleted by insertion of the kanamycin resistance gene (Km^r^). (F) Immunoblot analysis showing DnaA^WT^ and DnaA^R328H^ protein levels in culture under light and dark conditions. Proteins extracted from respective cells at the indicated time points were reacted with the anti-HA antibody (upper panel). CBB staining of the protein samples resolved by SDS-PAGE is shown in the lower panel as a gel loading control. (G) ChIP-qPCR analysis showing the affinity of DnaA (left) and DnaB (right) protein for the *oriC* region in DnaA^WT^ and DnaA^R328H^ cells under light and dark conditions. The DnaA (or DnaB)-chromatin complex was immunoprecipitated with anti-HA (or FLAG) antibody. For the DnaB assay, DnaB-FLAG was expressed under the control of the *dnaB* promoter (44). The samples were quantified by qPCR using primers specific to the *oriC* region and the *Syf1294* gene (1294), which is farthest from *oriC* in the circular chromosome. The value indicated is the ratio of percent recovery (*oriC*/1294) normalized to the value of DnaA^WT^ at hour 0 (defined as 1.0).

To address how replicating chromosome number is regulated, we examined the relationships of DnaA protein level and activity with chromosomal replication and chromosomal copy number in *S. elongatus*. It was previously shown that genomic DNA replication absolutely depends on photosynthetic activity in *S*. *elongatus*, so replication ceases under darkness (44). To examine whether DNA replication correlates with DnaA activity, we first compared DnaA protein level and *oriC*-binding activity between light and dark conditions. Cells were grown in an inorganic medium for 5 h under illumination and then kept under light or transferred to dark conditions (Fig. 2A). Immunoblot analysis using anti-DnaA antibody showed that DnaA protein level was constant for 3 h under both light and dark conditions (Fig. 2B and Fig. S5). In contrast, chromatin immunoprecipitation using the anti-DnaA antibody and subsequent quantitative PCR (ChIP-qPCR) analysis of the *oriC* region showed that the affinity of DnaA to *oriC* decreased by approximately two-thirds after 30 min under darkness (Fig. 2C). When an inhibitor of photosynthetic electron flow, 3-(3,4-dichlorophenyl)-1,1-dimethylurea (DCMU) or 2,5-dibromo-3-methyl-6-isopropyl-*p*-benzoquinone (DBMIB), was added to the culture under light, *oriC* binding of DnaA was almost completely abolished although DnaA level remained constant (Fig. S5 and Fig. S6A, B, and C). These results suggest that DNA replication activity is associated with the *oriC*-binding activity of DnaA rather than DnaA protein level. Further, *oriC* binding and DNA replication appear to depend on photosynthesis, which was confirmed in subsequent studies.

10.1128/mBio.00510-19.6 FIG S5 Comparison of DnaA expression levels in *S. elongatus* that was cultured under different conditions. Immunoblot analyses showing temporal changes in DnaA and SSB-GFP protein levels in *S. elongatus*. Mid-log-phase cultures of SSB-GFP cells were inoculated into fresh inorganic medium (hour 0) and then cultured for 9 h under the indicated illumination intensities and temperatures. Total proteins extracted from the cells at the indicated time points were reacted with the anti-DnaA antibody (upper panel) and anti-GFP antibody (lower panel). Samples at hour 0 are identical in the all blots. In order to clearly visualize the temporal change in protein levels for the 30°C and 250-µE m^−2^ s^−1^ treatment group, an image acquired with a shorter exposure time than the others is shown. Download FIG S5, EPS file, 0.5 MB. Copyright © 2019 Ohbayashi et al. 2019 Ohbayashi et al. This content is distributed under the terms of the Creative Commons Attribution 4.0 International license.

10.1128/mBio.00510-19.7 FIG S6 Effect of photosynthetic inhibitors on affinity of DnaA and DnaB proteins to genomic *oriC* region. (A) Schematic diagram of the culturing condition for panels B to E. A saturated cell culture of the *S. elongatus* wild type or HA-DnaA^R328H^ was inoculated into fresh inorganic medium and cultured with illumination (70 µE m^−2^ s^−1^) for 5 h. Then DCMU or DBMIB was added to the culture (hour 0), and cells were further cultured with illumination for 3 h. (B) Immunoblot analysis showing the DnaA protein level in a culture treated with DCMU or DBMIB under illumination. Total proteins extracted from cells at the indicated time points were reacted with the anti-DnaA antibody (upper panel). Coomassie brilliant blue (CBB) staining of the protein samples resolved by SDS-PAGE is shown in the lower panel as a loading control. (C and D) ChIP-qPCR analysis showing the effect of DCMU or DBMIB on the affinity of DnaA (C) and DnaB (D) for the *oriC* region. The DnaA- (or DnaB)-chromatin complex was immunoprecipitated with the anti-DnaA (or anti-FLAG) antibody. For the DnaB assay, a strain expressing DnaB-FLAG under the control of the *dnaB* promoter (44) was used. The samples were quantified by qPCR using the primers specific to the *oriC* region (*oriC*; gray bar) and the *Syf1294* gene (1294; white bar), which is furthest from *oriC* in the circular chromosome. The percent recovery against the amount of total input DNA is indicated. (E) ChIP-qPCR analysis showing the effect of DCMU on the affinity of HA-DnaA^R328H^ for the *oriC* region. The samples were subjected to ChIP-qPCR analysis with the anti-HA antibody. The value indicated is the ratio of percent recovery (*oriC*/1294). Download FIG S6, EPS file, 0.7 MB. Copyright © 2019 Ohbayashi et al. 2019 Ohbayashi et al. This content is distributed under the terms of the Creative Commons Attribution 4.0 International license.

### ATP-dependent binding of DnaA to *oriC* in *S. elongatus*.

In many model bacterial species, binding of DnaA to the *oriC* region is controlled by the ATP/ADP ratio, as only the ATP-bound form is capable of forming the oligomeric structure at the *oriC* region required for initiation of DNA replication (45). To assess the mechanism of light/photosynthesis-dependent activation of DnaA activity in *S*. *elongatus*, we examined the effect of changing the proportion of ATP-bound DnaA on *oriC*-binding activity under constant illumination.

The arginine residue at position 334 of E. coli DnaA is essential for ATP hydrolysis, and the amino acid substitution R334H inactivates this intrinsic ATPase activity, which leads to accumulation of DnaA-ATP (46). This arginine is conserved in DnaA of many organisms (47), including cyanobacteria (Fig. 2D). In order to examine the effect of ATP binding to DnaA on *oriC* affinity in *S. elongatus*, we expressed HA-DnaA (DnaA^WT^) or HA-DnaA R328H (DnaA^R328H^) (which corresponds to R334H of E. coli DnaA) under the control of the *dnaA* promoter from the genomic neutral site (NS I) on an endogenous *dnaA*-knockout background (Fig. 2E and Fig. S7). Binding to *oriC* was then examined by chromatin immunoprecipitation (ChIP). For these studies, HA tag was fused to the N terminus of DnaA^WT^ or DnaA^R328H^ so that the protein could be precipitated with an anti-HA antibody instead of anti-DnaA because the affinity of the polyclonal anti-DnaA antibody would likely differ between DnaA^WT^ and DnaA^R328H^.

10.1128/mBio.00510-19.8 FIG S7 Revision of the start codon of *S*. *elongatus dnaA* gene. (A) Amino acid sequence (deduced from the annotated ORF sequences) alignment of the DnaA N-terminal regions of *S*. *elongatus* (syf), *Synechococcus* sp. strain UTEX2973 (syu), *Cyanothece* sp. strain PCC 7425 (cyn), *Anabaena* sp. (ana), *Synechocystis* (syn), and Bacillus subtilis 168 (bus). (B) DNA sequence of *S*. *elongatus dnaA* gene around the annotated start codon (CyanoBase, http://genome.kazusa.or.jp/cyanobase). Based on the protein expression analyses in panel C, the GTG codon 60 bp downstream of the annotated translational start codon (ATG) turned out to be the actual start codon of the *dnaA* gene in *S*. *elongatus*. (C) Three constructs (a to c) for expression from the chromosomal neutral site I (NS I). In construct a, the HA-tag-encoding sequence was fused with the annotated start codon (ATG) of *dnaA* and expressed by the IPTG-inducible *trc* promoter. In construct b, the HA-tag-encoding sequence was fused with the revised start codon (GTG) of *dnaA* and expressed by the 60-bp 5′ upstream sequence flanking the revised start codon (GTG). In construct c, the HA-tag-encoding sequence was fused with the revised start codon (GTG) of *dnaA* and expressed by the 300-bp 5′ upstream sequence flanking the revised start codon (GTG). After introduction of each HA-tagged *dnaA*, the endogenous *dnaA* gene was deleted in all strains. (D) Immunoblot analysis showing the size of HA-DnaA expressed in the respective strains. Mid-log-phase cultures were inoculated into fresh medium with (+) or without (−) 1 mM IPTG. Total proteins extracted from respective transformants (for construct a, with or without IPTG induction) were subjected to immunoblot analysis with the anti-DnaA antibody (upper panel) and the anti-HA antibody (lower panel). In construct a, HA-DnaA protein was expressed only by IPTG induction, while the smaller endogenous DnaA protein was expressed both with and without IPTG, suggesting that this construct possesses a promoter other than the *trc* promoter. In addition, in constructs b and c, HA-DnaA was expressed by the predicted *dnaA* promoter (upstream of GTG; US60 or US300). The HA-DnaA expressed by constructs b and c was smaller than that expressed by construct a. These results indicate that the actual start codon of the *dnaA* gene is a “GTG” 60 bp upstream of the “GTG” functioning as a promoter of the *dnaA* gene. Download FIG S7, EPS file, 0.7 MB. Copyright © 2019 Ohbayashi et al. 2019 Ohbayashi et al. This content is distributed under the terms of the Creative Commons Attribution 4.0 International license.

Both DnaA^WT^ and DnaA^R328H^ proteins were expressed in respective transformants at similar levels, and the expression levels remained constant for 3 h after cells were transferred to darkness (Fig. 2F). Similarly to endogenous DnaA (Fig. 2C), DnaA^WT^ bound to the *oriC* region in cells cultured under illumination and the affinity was reduced when the cells were transferred to darkness (Fig. 2G). The R328H mutation elevated DnaA activity due to an approximately 6-fold increase in *oriC* affinity compared to DnaA^WT^ under illumination (Fig. 2G). When the DnaA^R328H^ cells were transferred to darkness, the affinity of DnaA^R328H^ to *oriC* decreased but the level was still higher than DnaA^WT^ (Fig. 2G). These results suggest that the ATP-bound form of DnaA possesses higher affinity for the *oriC* site than the ADP-bound form as shown in other model bacteria. In addition, the light/photosynthesis-dependent activation of DnaA is, at least partly, independent of DnaA intrinsic ATPase activity because the affinity of constitutive DnaA^R328H^ for *oriC* decreased when the cells were transferred to darkness or treated with DCMU (Fig. 2G and Fig. S6E). This assumption is based on the following observations in E. coli. The R334H mutation completely abolished the intrinsic ATPase activity of DnaA *in vitro* (46). However, ATP bound to the mutated DnaA was gradually hydrolyzed by the addition of crude extract of E. coli *in vitro* (46). In addition, ATP bound to DnaA was slowly hydrolyzed by ATPases other than DnaA *in vivo* (23).

To examine the effect of DnaA^R328H^ expression and light/photosynthesis on replication fork components other than DnaA, we compared affinity of DnaB to *oriC* between DnaA^WT^ and DnaA^R328H^ transformants by ChIP-qPCR analysis. DnaB is a DNA helicase recruited to the *oriC* region in a DnaA- and DnaC-dependent manner in bacteria. Similar to DnaA, the affinity of DnaB to *oriC* was higher in DnaA^R328H^ cells than in DnaA^WT^ cells under both light and dark conditions (Fig. 2G). In addition, the affinity of DnaB to *oriC* decreased in both DnaA^R328H^ and DnaA^WT^ cells after transfer to darkness. These results suggest that the increased affinity of DnaA to *oriC* by light/photosynthesis or by an increase in ATP-DnaA by the R328H mutation promoted the formation of replication forks.

The *oriC*-binding activity of DnaA^R328H^ was 6-hold higher than that of DnaA^WT^ under illumination, although the expression level of DnaA^R328H^ protein was comparable to that of DnaA^WT^ (Fig. 2G). Thus, it is suggested that DnaA activity is limited, probably by moderation of ATP hydrolysis, even under illumination to restrict the number of replicating chromosomes as shown below.

### Simultaneous replication of multiple chromosome copies by increasing ATP-DnaA.

**FIG 3:**
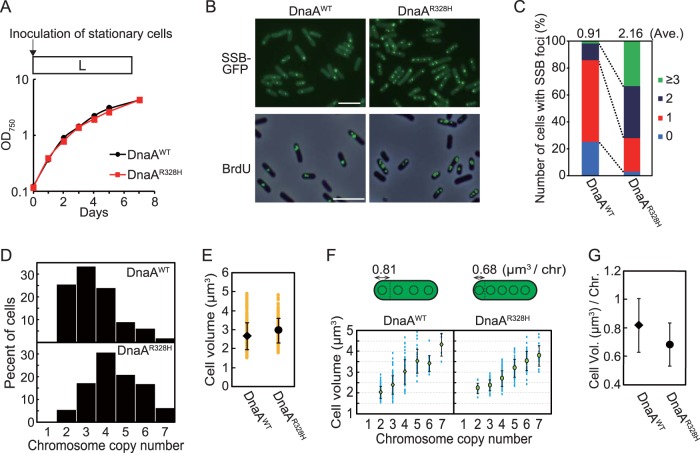
Chromosome replication in HA-DnaA^WT^ and HA-DnaA^R328H^ cells under illumination. (A) To visualize replicating chromosomes by fluorescence microscopy, GFP-tagged SSB protein was expressed in DnaA^WT^ and DnaA^R328H^ cells. Mid-log-phase cultures were inoculated into fresh inorganic medium (hour 0) and cultured under illumination. Growth curves of DnaA^WT^ (black) and DnaA^R328H^ (red) cells are shown. (B) Fluorescence microscopic images showing replicating chromosomes in DnaA^WT^ and DnaA^R328H^ cells. In the upper panel, SSB-GFP was observed in living DnaA^WT^ and DnaA^R328H^ cells at hour 6. In the lower panel, newly synthesized DNA was visualized by immunofluorescence microscopy with the anti-BrdU antibody. Cells were labeled with BrdU for 1 h (hour 5 to 6) and then fixed for immunofluorescence staining. The phase-contrast and immunofluorescence images are merged. Bar, 5 µm. (C) Frequencies of cells exhibiting zero (blue), one (red), two (deep blue), or three or more (green) SSB-GFP foci in DnaA^WT^ and DnaA^R328H^ cultures at hour 6 (*n *≥* *300 cells for each strain). The average number of SSB foci is indicated above the bars. (D) Histograms showing number of chromosomes per cell in DnaA^WT^ and DnaA^R328H^ cultures at hour 6. The number of chromosomes was determined based on micrographs of SYBR Green-stained cells. (E) Distribution of DnaA^WT^ and DnaA^R328H^ cell volumes at hour 6. Orange points represent volumes of single cells (*n *≥* *300 cells for each strain). The black diamond (DnaA^WT^) and circle (DnaA^R328H^) represent the means, and the error bars represent the standard deviations (*n *≥* *300 cells for each strain). (F) Distribution of DnaA^WT^ and DnaA^R328H^ cell volumes and chromosomal numbers at hour 6. Blue points represent single cells. The green diamond (DnaA^WT^) and circle (DnaA^R328H^) represent the means, and the error bars represent the standard deviations (*n *≥* *300 cells for each strain). (G) Mean cell volume occupied by one chromosome in DnaA^WT^ and DnaA^R328H^ cells at hour 6. The black diamond (DnaA^WT^) and circle (DnaA^R328H^) represent the means, and error bars represent the standard deviations (*n *≥* *300 cells for each strain).

As mentioned above, only one copy of multiple chromosomes is replicated in wild-type *S*. *elongatus* (37, 39, 40). This finding led us to examine the effect of artificially elevating DnaA activity by expressing DnaA^R328H^ on the number of replicating chromosome copies. To this end, we expressed GFP-tagged SSB to visualize replicating chromosomes in DnaA^WT^ and DnaA^R328H^ cells. Consistent with previous reports (37, 39, 40) and the results shown in Fig. 1, the majority of DnaA^WT^ cells exhibited only one SSB focus per cell during growth under illumination (Fig. 3A to C). In contrast, the majority of DnaA^R328H^ cells exhibited two or more SSB foci under the same growth conditions (Fig. 3B and C). When DnaA^WT^ and DnaA^R328H^ cells were labeled with BrdU, one or two BrdU foci were detected in DnaA^WT^ cells (Fig. 3B) whereas two or more foci were detected in DnaA^R328H^ cells 6 h after inoculation under illumination (Fig. 3B). These results indicate that elevation of DnaA activity by enhancing the ratio of ATP-DnaA to total DnaA results in simultaneous replication of multiple chromosome copies.

In order to compare frequency of DNA replication initiation between DnaA^WT^ and DnaA^R328H^ cells on a genome-wide basis, relative copy numbers of chromosomal regions in these strains were analyzed by next-generation sequencing. When model bacteria possessing a single chromosome (genome) per cell, such as E. coli, are rapidly grown in nutrient-rich medium, DNA is replicated in a multifork mode. In this case, the *oriC*/*terC* ratio is more than 2, thus yielding a V-shaped profile in the depth of sequencing reads (lowest at *terC* and highest at *oriC*) (37). In the case of cyanobacteria possessing multiple chromosome copies, of which only one copy is being replicated, the ratio of *terC* and *oriC* to total DNA is almost 1.0, thus yielding an almost linear profile in the depth of sequencing reads (37) (Fig. S8A, DnaA^WT^) throughout the genome. The sequencing profile of DnaA^R328H^ cells 6 h after inoculation under illumination exhibited a slight V-shape, in contrast to the sequencing profile of DnaA^WT^ cells (highest at *oriC* region; Fig. S8A). However, the ratio of *oriC* to *terC* was less than 2 (Fig. S8A). These results suggest that some chromosomes are replicated simultaneously in DnaA^R328H^ cells under illumination but that the number is still restricted, most likely by ATPase proteins other than DnaA. Nonetheless, this restriction was compromised to a certain extent when the intrinsic ATPase activity was abolished by the R328H mutation.

10.1128/mBio.00510-19.9 FIG S8 High-throughput sequencing of genomic DNA and chromosomal copy number in mid-log-phase cultures of DnaA^WT^ and DnaA^R328H^. (A) Depth of genomic DNA read through the genome 6 h after inoculation in DnaA^WT^ and DnaA^R328H^ strains was analyzed by Illumina MiSeq. Relative sequencing coverage ratios of Illumina MiSeq reads (1-kb window for the left graphs and 100-kb window for the right graphs) at respective genomic regions in HA-DnaA^WT^ and HA-DnaA^R328H^ cells are shown. The ratio of read depth is shown in the graphs. The sequencing coverage of each region was normalized to the total read depth. (B) Flow cytometry analysis showing the DNA level and relative cell volume of DnaA^WT^ (upper panel) and DnaA^R328H^ (lower panel) cells 6 h after inoculation. Dots represent single cells. DNA level was determined by intensity of SYTOX Green fluorescence. Download FIG S8, EPS file, 0.7 MB. Copyright © 2019 Ohbayashi et al. 2019 Ohbayashi et al. This content is distributed under the terms of the Creative Commons Attribution 4.0 International license.

### Relationship between cell growth and DNA replication in *S. elongatus*.

Chromosome copy number exhibits a positive, linear correlation with cell volume in *S*. *elongatus* (18, 39, 40). However, it is not known whether increased chromosomal copy number leads to increased cell volume or if cell growth leads to an increased number of chromosomal copies. To address whether there is such a causal relationship between chromosomal copy number and cell size, we examined the relationships among growth rate, cell volume, and frequency of chromosomal replication.

First, we investigated the effect of changing the chromosome copy number on cell growth and cell volume by comparing DnaA^R328H^ and DnaA^WT^ cells. When DnaA^R328H^ and DnaA^WT^ were cultured under illumination, DnaA^R328H^ cells possessed more chromosome copies than DnaA^WT^ cells (Fig. 3D). Thus, an increase in the number of replicating chromosome by expression of DnaA^R328H^ led to an increase in chromosomal copy number per cell. However, DnaA^R328H^ and DnaA^WT^ cells exhibited similar growth rates (Fig. 3A) and cell volumes (Fig. 3E). Thus, the cell volume occupied by one chromosome in DnaA^R328H^ cells was smaller than that in DnaA^WT^ cells (Fig. 3F and G). Similar results were also observed by measuring cell volume and DNA level by flow cytometry (Fig. S8B). These results suggest that initiation frequency and number of chromosome copies have little impact on cellular growth rate and volume.

**FIG 4:**
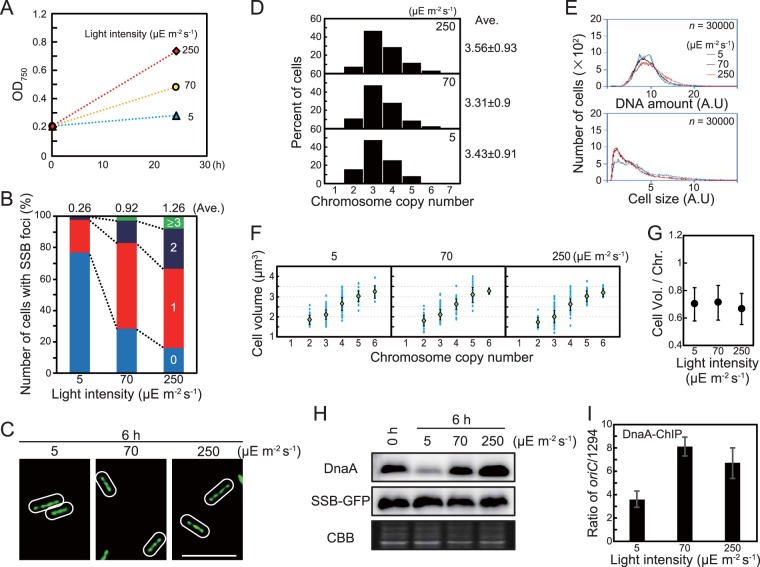
Effect of growth rate on number of replicating chromosomes, chromosome copy number, and cell size. To prepare cultures with different growth rates, *S. elongatus* was cultured under different intensities of illumination at 30°C. (A) Growth rate at 5, 70, and 250 µE m^−2^ s^−1^. A mid-log-phase SSB-GFP culture was inoculated into fresh inorganic medium (hour 0) and then cultured under the indicated illumination intensity. (B) Frequency of cells exhibiting zero (blue), one (red), two (deep blue), or three or more (green) SSB-GFP foci at hour 6 (*n *≥* *300 cells per condition). The average number of SSB foci is indicated above the bars. (C) Fluorescence microscopic images showing SYBR Green staining of nucleoids at hour 6. Phase-contrast and SYBR Green images are shown. Bar, 5 µm. (D) Histograms showing number of chromosomes per cell at hour 6. Number of chromosomes was determined based on micrographs of SYBR Green-stained cells (*n *≥* *300 cells per condition). (E) Flow cytometry analysis showing the DNA level (upper panel) and relative cell volume (lower panel) at hour 6. DNA level was calculated by intensity of SYTOX Green fluorescence. Blue line, 5 µE m^−2^ s^−1^; black line, 70 µE m^−2^ s^−1^; red line, 250 µE m^−2^ s^−1^; A.U., arbitrary unit. (F) Distribution of cell volume and chromosomal copy number. The blue points represent single cells. The green diamond represents the mean, and the error bar represents the standard deviation (*n *≥* *300 cells per condition). (G) Mean cell volume occupied by one chromosome at hour 6. Error bar represents the standard deviation (*n *≥* *300 cells per condition). (H) Immunoblot analysis showing DnaA and SSB-GFP protein levels. Proteins extracted from cells at hours 0 and 6 were reacted with anti-HA antibody (top) and anti-GFP antibody (middle). Coomassie brilliant blue (CBB) staining of the protein samples resolved by SDS-PAGE is shown in the bottom panel as a gel loading control. (I) ChIP-qPCR analysis showing the affinity of DnaA for the *oriC* region at hour 6. The value indicated is the ratio of percent recovery (*oriC*/1294). Error bars represent the standard deviation (*n *=* *3). Three independent experiments yielded similar results, and results from one experiment are shown (for panels A to H).

Next, to examine the effect of cell growth on chromosomal replication and chromosome copy number, we cultured *S*. *elongatus* under different light intensities (Fig. 4A). *S. elongatus* is an obligate photoautotroph, so growth rate depends on light intensity. Cultures expressing SSB-GFP exhibited faster growth at higher light intensity [0.01 h^−1^ at 5 µE (µmol photons) m^−2^ s^−1^, 0.03 h^−1^ at 70 µE m^−2^ s^−1^, and 0.05 h^−1^ at 250 µE m^−2^ s^−1^] (Fig. 4A). As light intensity and growth rate increased, the number of SSB foci per cell also increased (average of 0.26 foci per cell at 5 µE m^−2^ s^−1^, 0.92 at 70 µE m^−2^ s^−1^, and 1.26 at 250 µE m^−2^ s^−1^) (Fig. 4B). On the other hand, cells growing under different light intensities exhibited similar chromosome copy numbers and cell volumes and thus similar positive correlations between cell volume and chromosomal copy number (Fig. 4C to G). Comparable results were also observed by measuring cell volume and DNA level by flow cytometry (Fig. S9A). Changing the light intensity also influenced DnaA level. Specifically, DnaA level decreased when light intensity was reduced to 5 µE m^−2^ s^−1^ (hour 6) from 70 µE m^−2^ s^−1^ (hour 0) (Fig. 4H and Fig. S5) and increased when the light intensity was raised to 250 µE m^−2^ s^−1^ (hour 6) from 70 µE m^−2^ s^−1^ (hour 0) (Fig. 4H and Fig. S5). In addition, the *oriC*-binding activity of DnaA was lower at 5 µE m^−2^ s^−1^ than at 70 or 250 µE m^−2^ s^−1^ (Fig. 4I; note that in the ChIP-qPCR analysis, sample input DnaA depends on the cellular DnaA level). These results suggest that an increase or decrease in cellular growth rate leads to a corresponding increase or decrease in chromosomal replication activity to maintain a constant chromosomal copy number per unit cell volume at a constant temperature.

**FIG 5:**
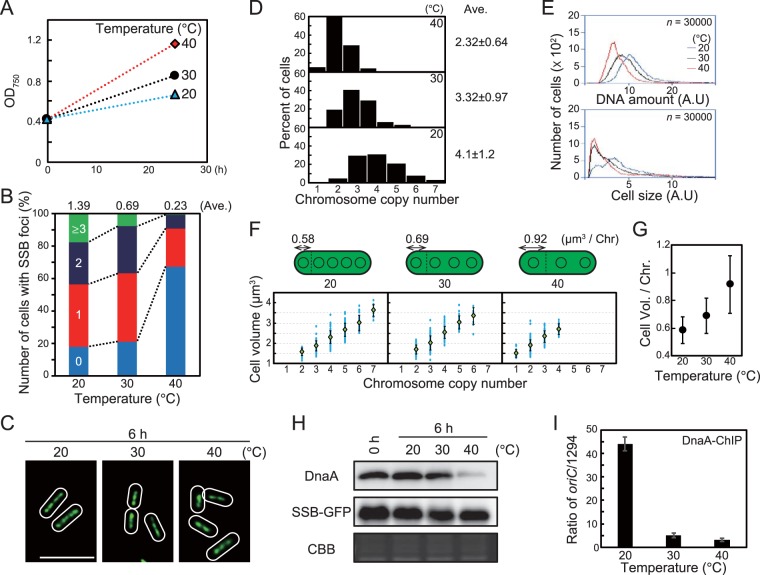
Effect of temperature on growth rate, number of replicating chromosomes, chromosome copy number, and cell size. (A) Growth rate of *S. elongatus* at 20, 30, and 40°C. A mid-log-phase SSB-GFP culture at 30°C was inoculated into fresh inorganic medium (hour 0) and then cultured at the indicated temperature under 70-µE m^−2^ s^−1^ illumination. (B) Frequency of cells exhibiting zero (blue), one (red), two (deep blue), or three or more (green) SSB-GFP foci at hour 6 (*n *≥* *300 cells per condition). The average number of SSB foci is indicated above the bars. (C) Fluorescence microscopic images showing SYBR Green staining of nucleoids at hour 6. SYBR Green images are shown. Bar, 5 µm. (D) Histograms showing number of chromosomes per cell at hour 6. Number of chromosomes was determined based on micrographs of SYBR Green-stained cells (*n *≥* *300 cells per condition). (E) Flow cytometry analysis showing the DNA level (upper) and relative cell volume (lower) at hour 6. DNA level was calculated by intensity of SYTOX Green fluorescence. Blue line, 20°C; black line, 30°C; red line, 40°C; A.U., arbitrary unit. (F) Distribution of cell volume and chromosomal copy number. The blue points represent single cells. The green diamond represents the mean, and the error bar represents the standard deviation (*n *≥* *300 cells per condition). The schematic illustrations above the graphs show the average cell volume occupied by one chromosome. (G) Mean cell volume occupied by one chromosome at hour 6. Error bar represents the standard deviation (*n *≥* *300 cells per condition). (H) Immunoblot analysis showing DnaA and SSB-GFP protein levels. Proteins extracted from cells at hour 0 and 6 were reacted with the anti-HA antibody (top) and anti-GFP antibody (middle). Coomassie brilliant blue (CBB) staining of the protein samples resolved by SDS-PAGE is shown in the bottom panel as a gel loading control. (I) ChIP-qPCR analysis showing the affinity of DnaA for the *oriC* region at hour 6. The value indicated is the ratio of percent recovery (*oriC*/1294). Error bars represent the standard deviation (*n *=* *3). Three independent experiments yielded similar results. Results from one experiment are shown (for panels A to H).

10.1128/mBio.00510-19.10 FIG S9 Flow cytometry analysis of cell volume and DNA level of cells grown under different illumination intensities or temperatures. Flow cytometry analysis showing the DNA level and cell volume as in Fig. 4A (A) and Fig. 5E (B). Dots represent single cells. DNA level was determined by intensity of SYTOX Green fluorescence. Download FIG S9, EPS file, 0.5 MB. Copyright © 2019 Ohbayashi et al. 2019 Ohbayashi et al. This content is distributed under the terms of the Creative Commons Attribution 4.0 International license.

When the cells were transferred from light to dark or photosynthesis was inhibited with DCMU or DBMIB, cellular growth and chromosome replication ceased whereas DnaA level remained constant (Fig. 2 and Fig. S5 and S6). In contrast, when the light intensity was reduced and cellular growth slowed down, DnaA level decreased (Fig. 4 and Fig. S5). Thus, DnaA level changes only when cells are growing and the growth rate changes to match chromosome replication rate, but does not change when cellular growth and chromosome replication cease.

We also examined the relationship between cell growth and chromosomal replication and copy number at different temperatures. As temperature increased, cell growth accelerated (0.01 h^−1^ at 20°C, 0.03 h^−1^ at 30°C, and 0.05 h^−1^ at 40°C; Fig. 5A). In contrast to results at constant temperature (30°C) and variable light intensity (Fig. 4), the number of SSB foci per cell increased at lower temperature (Fig. 5B) even though cell growth was slower (Fig. 5A). Consistent with these observations, microscopic analysis and flow cytometry showed that chromosome copy number per cell was higher at lower temperature 6 h after temperature reduction (Fig. 5C, D, and E) while cell volume per chromosome was reduced at lower temperature (Fig. 5F and G). Similar results were also observed by measuring cell volume and DNA level by flow cytometry (Fig. S9B). The level and *oriC*-binding activity of DnaA were higher at lower temperature (Fig. 5H and I; note that in the ChIP-qPCR assay, sample input DnaA level depends on cellular DnaA level). Although cell volume per chromosome depended on temperature, cell volume and chromosomal copy number exhibited a linear correlation at all temperatures examined. These results suggest that the number of replicating chromosomes (initiation frequency) and chromosomal copy number are regulated by a certain temperature-affected factor.

### Multicopy chromosomes confer greater UV resistance to *S. elongatus*.

Photoautotrophic organisms require light for growth. However, UV in sunlight damages DNA directly and the photosystems generate reactive oxygen species (ROS) that also damage DNA (48). Thus, photosynthetic organisms are exposed to higher risk of DNA damage during cell growth than many other heterotrophic organisms. Polyploid bacteria often inhabit extreme environments such those with high temperatures (e.g., Thermus thermophilus) and possess resistance to high UV (e.g., Deinococcus radiodurans). In a similar manner, polyploid chloroplasts and mitochondrial DNA are also exposed to oxidative stresses that result from photosynthesis and respiration (49, 50). Thus, one plausible advantage of multiple chromosome copies is to compensate for a damaged copy using the information from the undamaged replicate.

**FIG 6:**
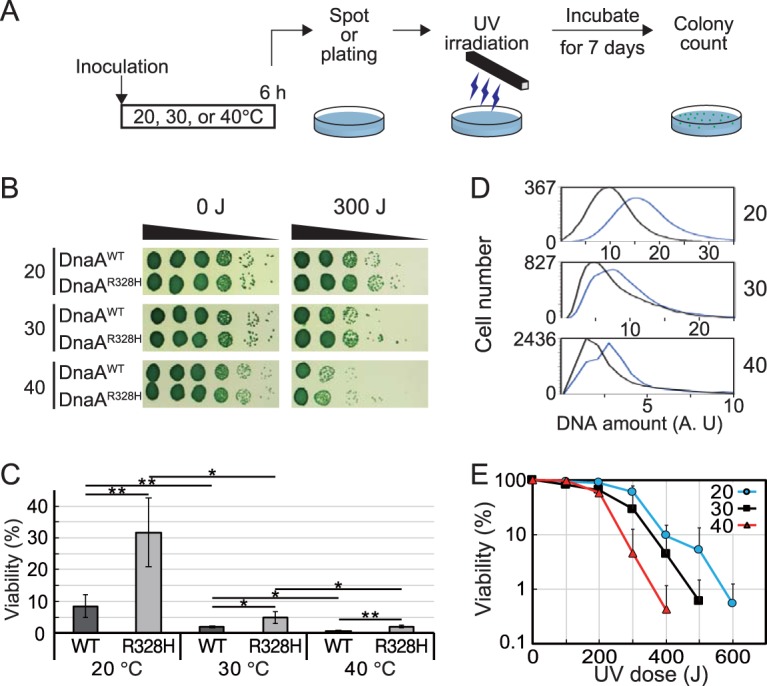
Effect of polyploidy level on UV resistance of the cells. (A) Schematic diagram of the assay. A mid-log-phase DnaA^WT^ or DnaA^R328H^ culture was inoculated into fresh inorganic medium and then cultured for 6 h at the indicated temperature under 70-µE m^−2^ s^−1^ illumination. Cell density was adjusted to an OD_750_ of 0.2, and a serial dilution series (10^−1^ to 10^−5^) was prepared using fresh medium. The diluted cells were spotted (for panel B) and plated (for panels C and D) on agar medium and then irradiated under 0- to 600-J m^−2^ UV-C (254 nm), respectively. The plates were further incubated at 30°C under illumination (70 µE m^−2^ s^−1^) for 7 days to allow surviving cells to form colonies. (B and C) The cell survival rate was calculated by counting the colony formation units. Results of DnaA^WT^ and DnaA^R328H^ cells cultured at 20°C, 30°C, or 40°C under 300-J m^−2^ UV-C irradiation are shown. Error bars represent the standard deviation (*n *=* *3 biological replicates). Two-tailed *t* tests between the indicated strains or conditions were performed. *, *P < *0.05; **, *P < *0.01. (D) Flow cytometry analysis showing the DNA level at hour 6 in DnaA^WT^ and DnaA^R328H^ cells cultured at 20°C, 30°C, or 40°C. Black line, DnaA^WT^; blue line, DnaA^R328H^; A.U., arbitrary unit. (E) The survival rate of the wild-type cells was determined after irradiation with 0- to 600-J m^−2^ UV-C at 20, 30, or 40°C. Error bars represent the standard deviation (*n *=* *3 biological replicates).

In order to assess this possibility, we investigated the relationship between ploidy level and cellular resistance to UV in *S. elongatus*. Populations with different ploidy levels were generated by cultivation of DnaA^WT^ cells and DnaA^R328H^ cells at 20, 30, or 40°C (Fig. 3 and 5). After cultivation in liquid culture under light for 6 h, cells were spotted onto agar medium. After UV irradiation, cells were cultured on agar medium at 30°C to allow surviving single cells to produce colonies (Fig. 6A and B). Cell viability just after UV irradiation was quantified based on the number of single colonies (Fig. 6C and E). As temperature increased and ploidy level decreased (Fig. 5), susceptibility to UV increased in the wild type (Fig. 6E). Ploidy was higher in DnaA^R328H^ than DnaA^WT^ cells at respective temperatures, and resistance to UV was also greater in DnaA^R328H^ cells than in DnaA^WT^ cells at respective temperatures (Fig. 6C and D). Thus, cells of higher ploidy were more resistant to UV irradiation, supporting the hypothesis that polyploidy benefits organisms under higher risk of DNA damage.

## DISCUSSION

### DnaA-dependent regulation of multicopy chromosomes in cyanobacteria.

Our results suggest that DNA replication in *S. elongatus* is absolutely dependent on photosynthesis through regulation of DnaA activity (Fig. 2; see also Fig. S6 in the supplemental material) and that the number of replicating chromosomes is restricted so that chromosome replication frequency is matched to cellular growth rate. Through this coordination, chromosome copy number increases and decreases according to cell size (Fig. 4 and 5), thereby maintaining protein and metabolite concentrations during cell growth.

Supporting this conclusion, inhibition of DnaA intrinsic ATPase activity by the R328H mutation increased the number of replicating chromosomes during cell growth (Fig. 3). However, DNA replication was still limited to a certain chromosome copy number in DnaA^R328H^ cells (Fig. 3; Fig. S8). In addition, the *oriC*-binding activity of DnaA^R328H^ decreased when photosynthesis and cell growth were blocked by transfer to darkness or chemical inhibition of photosynthesis, although DnaA^R328H^ cells still exhibited higher *oriC*-binding activity than DnaA^WT^ cells (Fig. 2; Fig. S6). These results suggest that in addition to the intrinsic ATPase activity of DnaA, other, as-yet-unknown factors also regulate DnaA activity in *S. elongatus*. There are several steps during initiation in which regulatory systems have been found to control bacterial DNA replication (for a review, see reference 23). For example, *oriC* binding of ATP-DnaA is inhibited by SeqA in E. coli, Spo0A in B. subtilis, and CtrA in C. crescentus, all of which occupy the *oriC* region.

In addition, there are also likely DnaA-independent mechanisms that regulate the number of replicating chromosomes in cyanobacteria. In this study, we showed that the number of replicating chromosome is also restricted to one in *Synechocystis* cells during growth (Fig. 1). As in *S. elongatus*, chromosome replication in *Synechocystis* depends on photosynthesis (44). However, even when DnaA protein is depleted, *Synechocystis* grows in a manner similar to the wild type, and it is likely that initiation and regulation of chromosome replication in *Synechocystis* are governed mainly by unknown DnaA-independent mechanisms (38) that also function in *S. elongatus*.

### Relationship between cell growth and initiation of chromosome replication.

In this study, we showed that DnaA level, DnaA activity, and number of replicating chromosomes changed depending on cellular growth rate (Fig. 4 and 5). In addition, chromosome copy number per unit cell volume was nearly constant during cell growth at a constant temperature (Fig. 4 and 5). On the other hand, increasing the number of replicating chromosomes and chromosome number per cell by inhibition of DnaA ATPase activity did not affect cell growth (Fig. 3). These results suggest that the number of replicating chromosomes is regulated by cell growth through regulation of DnaA level and activity in *S*. *elongatus.*

**FIG 7:**
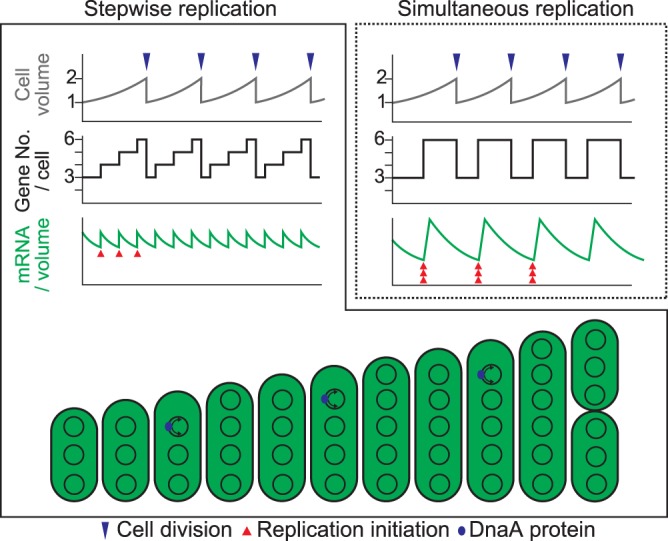
Correlation between cell growth and chromosomal replication in *S*. *elongatus*. Schematic diagrams showing changes in cell volume (top), gene copy number per cell (middle), and mRNA level per unit volume (bottom) during cell growth in the case of stepwise (as observed in this study) or simultaneous replication of multicopy chromosomes. Blue and red arrowheads indicate cell division and replication of a chromosomal copy, respectively.

Regulation of chromosome replication depending on cellular growth has also been demonstrated in monoploid bacteria such as E. coli, in which chromosome replication is initiated when the cell grows to a certain fixed volume (51, 52). However, in both monoploid and polyploid bacteria, it is still unclear how a cell senses its volume to regulate chromosome replication. When cell volume increases, concentrations of RNAs transcribed from chromosomes and proteins translated from these mRNAs are predicted to decrease until additional chromosome copies are generated by replication (Fig. 7). Therefore, chromosomal replication is likely triggered by a reduction in the concentration of an as-yet-unknown factor or factors by cell growth. For example, chromosomal replication may be initiated when the concentration of a replication repressor decreases below a certain threshold. This notion is supported by the observation that DnaA level and activity, number of replicating chromosomes, and chromosome number per cell volume were lower at higher temperature in *S. elongatus* (Fig. 5). Increased temperature accelerates chemical reactions, including enzymatic reactions; thus, transcriptional and translational activities increase with temperature. At higher temperature, cells require lower levels of template chromosomal DNA and thus a smaller number of chromosomal copies to sustain protein and metabolite levels than at lower temperature.

### Biological significance of polyploidy in bacteria.

Polyploidy is widespread in bacteria, archaea, and eukaryotes, but the advantages conferred by multicopy genomes are unclear. Results of this study suggest that polyploid bacteria are able to change chromosomal copy number per cell during cell growth to maintain nearly constant gene copy number per unit cell volume (Fig. 7) and that multiple copies of chromosomes in bacteria act as backup genetic information to compensate for damage to the other chromosomal copies. In accord with the first notion, compared to monoploid organisms or polyploid organisms exhibiting simultaneous replication of chromosomes, in which gene number per unit cell volume changes up to 2-fold (53), stepwise replication of chromosomal copies in polyploids such as *S*. *elongatus* reduces changes in the gene number/cell volume ratio during cell growth and division. Thus, the stepwise replication of multiple chromosomal copies enables a cell to keep nearly constant chromosome number per unit cell volume, which coordinates chromosomal number with cell size (Fig. 7) and probably contributes to maintaining a constant mRNA and protein concentration during cell growth as observed in *S. elongatus* (18). Consistent with chromosome copies as backup genetic information, some known polyploid eukaryotes inhabit extreme environments (7, 12–16) that can damage DNA (48). In addition, cyanobacterial growth depends on sunlight, which contains UV, and photosynthesis generates ROS, and both UV radiation and ROS damage DNA (48, 54). In this study, *S. elongatus* cells of higher ploidy exhibited greater UV resistance. Thus, despite the higher metabolic cost of polyploidy than of monoploidy, possessing multiple copies of a chromosome that replicates asynchronously probably benefits the organism by compensating for a damaged copy.

## MATERIALS AND METHODS

### Strains and culture conditions.

Strains used in this study were Synechococcus elongatus PCC 7942 (WT) and several transformants (described below) as well as *Synechocystis* sp. PCC 6803 (GT-I strain) (55) and several transformants (described below) including a thymidine kinase (TK) strain (38) expressing thymidine kinase for the BrdU assay and a strain expressing DnaB-FLAG under the control of the *dnaB* promoter (44). All cyanobacterial strains were cultured at 30°C unless otherwise indicated in BG-11 liquid medium with air bubbling and illumination of 70 µE m^−2^ s^−1^ unless otherwise indicated. DCMU or DBMIB was added to the culture at a final concentration of 5 µM to inhibit photosynthetic electron flow.

### ChIP-qPCR analyses.

Combined chromatin immunoprecipitation and qualitative PCR (ChIP-qPCR) analyses were performed according to the method of Hanaoka and Tanaka (56) with minor modifications. Cells were fixed with 1% formaldehyde for 15 min at room temperature. The cross-link reaction was stopped by the addition of glycine (final concentration of 125 mM) and incubation at room temperature for 5 min. Cells were harvested by centrifugation, washed twice with ice-cold Tris-buffered saline (TBS) (20 mM Tris-HCl, pH 7.4, 150 mM NaCl), and stored at −80°C. Fixed cells were broken using Beads Crusher (Taitec) with glass beads (<106 μm; Sigma Aldrich) in TBS at 4°C, and the genome was fragmented to approximately 500 bp using a Covaris sonication system (MS Instrument Inc.). After centrifugation for 15 min to precipitate the insoluble fraction, the supernatant containing genomic DNA was subjected to immunoprecipitation using an anti-HA antibody (clone 16B12; BioLegend), anti-SyfDnaA antibody (38), or anti-FLAG M2 antibody (F1804; Sigma) at a dilution of 1:250. Precipitated DNA was quantified by qPCR using primer sets oriC-F and oriC-R for the *oriC* region and 1294-F and 1294-R for the genomic region farthest from *oriC* in the circular genome. Primer sequences are listed in Text S1 in the supplemental material.

10.1128/mBio.00510-19.1 TEXT S1 Supplemental methods. Contains Table S1 (primers used in this study). Download Text S1, DOCX file, 0.7 MB. Copyright © 2019 Ohbayashi et al. 2019 Ohbayashi et al. This content is distributed under the terms of the Creative Commons Attribution 4.0 International license.

### Immunofluorescence microscopy.

Cells were fixed with 1% paraformaldehyde and 10% dimethyl sulfoxide dissolved in methanol at −30°C for 5 min. After washing twice with phosphate-buffered saline (PBS; 137 mM NaCl, 2.7 mM KCl, 10 mM Na_2_HPO_4_, 1.76 mM KH_2_PO_4_, pH 7.4), fixed cells were treated with 0.05% Triton X-100 in PBS for 15 min at room temperature. After washing twice with PBS, the fixed cells were further permeabilized with 0.2 mg ml^−1^ lysozyme (dissolved in 25 mM Tris-HCl, pH 7.5, 10 mM EDTA) at 37°C for 30 min. After two additional washes with PBS, genomic DNA was digested *in situ* with 4 M HCl at 37°C for 1 h. After washing twice with PBS, cells were blocked with Blocking One (Toyobo) at room temperature for 30 min and then were reacted with the anti-BrdU antibody (clone BMC9318; Roche) diluted in Blocking One (1:20) at 37°C overnight. After washing twice with PBS, cells were reacted with Alexa Fluor 488 goat anti-mouse antibody (Invitrogen) diluted in Blocking One (1:200). After two final washes with PBS, cells were observed by fluorescence microscopy.

### DNA staining for flow cytometry and observation of nucleoids by fluorescence microscopy.

Cells were harvested by centrifugation, fixed with 1% glutaraldehyde for 10 min, and washed with PBS. Fixed cells were stained with SYBR Green I (Invitrogen) (final concentration was 1:1,000) to count chromosomal copy number by fluorescence microscopy or with 10 µM Sytox Green for flow cytometry analysis. After staining, cells were subjected to flow cytometry (BD Accuri C6) as previously described (37).

### UV irradiation and evaluation of cell viability by spotting assay.

Mid-log-phase cultures of *S*. *elongatus* DnaA^WT^ or DnaA^R328H^ at 30°C were inoculated into fresh inorganic medium (BG-11) and cultured under 70-µE m^−2^ s^−1^ illumination at 20, 30, or 40°C. A portion of the culture was harvested 6 or 24 h after the inoculation and then adjusted to an OD_750_ of 0.2 for preparation of a serial dilution series (10^−1^ to 10^−5^) using fresh medium. The diluted cells were spotted on BG-11 agar plates and then irradiated with 0 to 600 J m^−2^ UV-C (254 nm) under a UV lamp (UVP; Upland). The plates were further incubated at 30°C under 70-µE m^−2^ s^−1^ illumination for 7 days, and cell survival rate was calculated by counting the colonies.
