# Supplementary material for: Coordination of Polyploid Chromosome Replication with Cell Size and Growth in a Cyanobacterium
Source: mBio. 2019 Apr 23;10(2):e00510-19. doi: 10.1128/mBio.00510-19 (PMC6478999; doi:10.1128/mBio.00510-19)
Supplement: TEXT S1 [file mBio.00510-19-s0001.docx]

**Supplemental Text**

**Supplementary Materials and Methods.**

**Immunoblotting**

Total cellular protein was separated using 8%, 10%, or 12% acrylamide gel at 20 μg per gel lane and then transferred to PVDF membranes. Membranes were blocked with 5% skim milk in TBS-T (10 mM Tris-HCl, pH 7.5, 150 mM NaCl, 0.1% Tween 20) and incubated in the one of the following primary antibodies at 1:1000: anti-HA antibody (clone 16B12, BioLegend), anti-SyfDnaA antibody (1), or the anti-GFP antibody (JL-8, Clontech). A HARP-conjugated anti-mouse IgG antibody (Thermo Scientific) was used as a secondary antibody at a dilution of 1:20,000. The signal was detected by ECL Prime Western Blotting Detection Regent (GE Healthcare) using an Image Quant LAS 4000 mini system (GE Healthcare).

**Analyses of chromosomal replication state by high-throughput sequencing**

Genomic DNA was extracted from DnaA^WT^ and DnaA^R328H^ cells. A 5-µg DNA sample from each group was fragmented to ~500 bp using a Covaris S2 sonication system (Covaris, Inc., Woburn, MA, USA). Sequencing libraries were prepared using the NEBNext DNA library prep kit for Illumina (New England Biolabs). Paired-end sequencing (320 cycles) was conducted using the MiSeq system (Illumina) according to the manufacturer’s specifications. The sequencing reads (total reads: 450,492,087 bases for DnaA^WT^ and 433,794,424 bases for DnaA^R328H^) were trimmed using CLC Genomics Workbench ver. 8.5.1 (Qiagen) with the following parameters: Phred quality score > 30, removal of the terminal 15 nucleotides from the 5’ end and 2 nucleotides from the 3’ end, and removal of truncated reads less than 100 nucleotides in length. Trimmed reads were mapped to the reference genome sequence of the chromosome and the plasmid of *Synechococcus elongatus* PCC 7942 (Accession number: NC_007604 and NC_007595) using CLC Genomics Workbench ver. 9.5.1 (Qiagen) with the following parameters: Length fraction: 0.7 and Similarity fraction: 0.9. To call SNPs and indels, we used filter settings as follows: minimum read depth for the SNP/indel calling = 10, minimum read depth for the SNP calling = 5, and 80% cutoff of percent aligned reads calling the SNP per total mapped reads at the SNP sites.

**Plasmid construction and preparation of stable *S. elongatus* transformants**

To produce the *S. elongatus* strain expressing HA-DnaA (Fig. S6), three constructs for expression of protein from the chromosomal neutral site I (NS I) were prepared (constructs a−c shown in Fig. S6C). The annotated *dnaA* *orf* (form annotated start codon “ATG”; Cyanobase, <http://genome.kazusa.or.jp/cyanobase> (2)) was amplified by PCR with primers 1 and 2, and the PCR product was cloned between *BamH*I and *Sal*I sites of pNSHA (possessing NS I sequences for double crossover recombination, *trc* promoter, and HA coding sequence; conferring spectinomycin resistance). To prepare constructs b and c (Fig. S6C), the 60-bp upstream sequence flanking the revised start codon (US60; revised start codon is “GTG”) was removed from construct ‘a’ using the Mutagenesis Kit (TOYOBO) with primers 3 and 4. After removal, a 60-bp upstream sequence (US60; amplified by PCR with primers 7 and 9) or 300-bp upstream sequence (US300; amplified by PCR with primers 8 and 9) flanking the revised *dnaA* start codon (GTG) was cloned into the vector, which was then amplified as a linear DNA by PCR with primers 5 and 6 using the In-Fusion Cloning Kit (TAKARA). Construct b was modified with primers 10 and 11 using the Mutagenesis Kit (TOYOBO) to introduce the *dnaA* R328H mutation.

To produce the *S. elongatus* strain expressing HA-tagged Herpes Simplex Virus type 1 TK from the chromosomal neutral site II, the following construct was prepared for transformation. The chromosomal neutral site II (NS II) was amplified by PCR with primers 12 and 13 and the product was cloned between *Eco*RI and *Bam*HI sites of pUC19. The *trc* promoter and HA coding sequence was amplified from pNSHA with primers 14 and 15. The *cat* gene (conferring chloramphenicol resistance) was amplified by PCR with primers 16 and 17 using pAM990 (3) as the template. The two PCR fragments were cloned into the *Nco*I site of the NS II using the In-Fusion HD Cloning Kit (TAKARA). Then, *Spe*I and *Xho*I sites were introduced into the vector just after the HA coding sequence with primers 18 and 19 using the Mutagenesis Kit (TOYOBO). Finally, the *TK* gene was amplified with primers 20 and 21 using pNSHA-TK (4) as the template and then cloned between *Spe*I and *Xho*I sites of the vector.

To produce the *S. elongatus* strain expressing SSB-GFP under control of the *ssb* promoter (Fig. S1), the following PCR products were prepared. The *ssb* *orf* (*Synpcc7942_0301*) flanked by the 466-bp upstream sequence was amplified by primers 22 and 23, *gfp* *orf* by primers 24 and 25 using pVZR-GFP as the template, the gentamicin resistant gene *Gm^r^* from pYCSFX (5) by primers 26 and 27, and the 480-bp 3’ downstream flanking sequence of *ssb* *orf* by primers 28 and 29. Amplified fragments were mixed and fused by recombinant PCR using primers 22 and 29, and the fused product was used for transformation. The insertion of *gfp* and *Gm^r^* genes into the chromosomal *ssb* locus was confirmed by PCR using primers 22 and 29 as shown in Fig. S1B.

To produce the *S. elongatus* strain expressing RpoC2-GFP under control of the *rpoC2* promoter (Fig. S3), the following PCR products were prepared. The 994-bp genomic region encoding the C-terminal fragment of RpoC2 (*Synpcc7942*_*1524*) was amplified using primers 36 and 37, *gfp*-*Gm^r^* fusion was amplified from genomic DNA of *S*. *elongatus ssb-gfp* strain using primers 38 and 39, and the 943-bp 3’-downstream flanking sequence of *rpoC2 orf* was amplified by PCR using primers 40 and 41. Amplified fragments were mixed and fused by recombinant PCR using primers 36 and 41, and the fused product was used for transformation. The insertion of the *gfp* and *Gm^r^* genes into the chromosomal *rpoC2* locus was confirmed by PCR using primers 42 and 43 as shown in Fig. S3B.

These vectors or the PCR product were transformed into *S. elongatus.* In the case of HA-DnaA expressers, the endogenous *dnaA* gene was subsequently deleted by the deletion cassette (1).

**Preparation of the stable transformant of *Synechocystis***

To produce the *Synechocystis* strain expressing SSB-GFP under control of the *ssb* promoter (Fig. S2), the following PCR products were prepared. The *ssb* *orf* (*slr0925*) flanked with the 700-bp upstream sequence was amplified using primers 30 and 31, *gfp*-*Gm^r^* fusion was amplified from genomic DNA of *S*. *elongatus ssb-gfp* strain by primers 32 and 33, and the 682-bp 3’-downstream flanking sequence of *ssb orf* was amplified using primers 34 and 35. Amplified fragments were mixed and fused by recombinant PCR using primers 30 and 35, and the fused product was used for transformation. Insertion of the *gfp* and *Gm^r^* genes into the chromosomal *rpoC2* locus was confirmed by PCR using primers 30 and 35 as shown in Fig. S2B.

To produce the *Synechocystis* strain expressing RpoC2-GFP by the *rpoC2* promoter (Fig. S4), the following PCR products were prepared. The 876-bp C-terminal-fragment-encoding sequence of *rpoC2* (*sll*_*1789*) was amplified using primers 44 and 45, the *gfp* gene fragment was amplified from genomic DNA of *S*. *elongatus ssb-gfp* strain using primers 46 and 47, the spectinomycin-resistant gene fragment *spec^r^* was amplified from genomic DNA of *S*. *elongatus* HA-DnaA^WT^ using primers 48 and 49, and the 799-bp 3’-downstream flanking sequence of *rpoC2 orf* was amplified using primers 50 and 51. These four PCR products were cloned into the plasmid pUC19, which was amplified as linear DNA by PCR using the primers 52 and 53 and the In-Fusion HD Cloning Kit (TAKARA). Insertion of the *gfp* and spectinomycin resistance genes into the chromosomal *rpoC2* locus was confirmed by PCR using primers 44 and 51 as shown in Fig. S4B.

**Table S1 Primers used in this study**

**References**

1. Ohbayashi R, Watanabe S, Ehira S, Kanesaki Y, Chibazakura T, Yoshikawa H. 2016. Diversification of DnaA dependency for DNA replication in cyanobacterial evolution. The ISME journal 10:1113-1121.

2. Fujisawa T, Narikawa R, Maeda S-i, Watanabe S, Kanesaki Y, Kobayashi K, Nomata J, Hanaoka M, Watanabe M, Ehira S. 2016. CyanoBase: a large-scale update on its 20th anniversary. Nucleic acids research 45:D551-D554.

3. Li R, Golden SS. 1993. Enhancer activity of light-responsive regulatory elements in the untranslated leader regions of cyanobacterial psbA genes. Proceedings of the National Academy of Sciences 90:11678-11682.

4. Watanabe S, Ohbayashi R, Shiwa Y, Noda A, Kanesaki Y, Chibazakura T, Yoshikawa H. 2012. Light‐dependent and asynchronous replication of cyanobacterial multi‐copy chromosomes. Molecular microbiology 83:856-865.

5. Fujita Y, Bauer CE. 2000. Reconstitution of light-independent protochlorophyllide reductase from purified BchL and BchN-BchB subunits in vitro confirmation of nitrogenase-like features of a bacteriochlorophyll biosynthesis enzyme. Journal of Biological Chemistry 275:23583-23588.
